# Undergraduate educational environment, perceived preparedness for postgraduate clinical training, and pass rate on the National Medical Licensure Examination in Japan

**DOI:** 10.1186/1472-6920-10-35

**Published:** 2010-05-20

**Authors:** Yasuharu Tokuda, Eiji Goto, Junji Otaki, Joshua Jacobs, Fumio Omata, Haruo Obara, Mina Shapiro, Kumiko Soejima, Yasushi Ishida, Sachiko Ohde, Osamu Takahashi, Tsuguya Fukui

**Affiliations:** 1 Mito Medical Center, University of Tsukuba Hospital, Institute of Clinical Medicine, Graduate School of Comprehensive Human Sciences, University of Tsukuba, Ibaraki, Japan; 2 Department of Medical Education, Yokohama City University School of Medicine, Yokohama, Kanagawa, Japan; 3 Department of Medical Education, Tokyo Medical University, Shinjuku, Tokyo, Japan; 4 University of Hawaii John A Burns School of Medicine, Honolulu, Hawaii, USA; 5 Center for Clinical Epidemiology, St. Luke's Life Science Institute, St. Luke's International Hospital, Tokyo, Japan; 6 Department of Medicine, Okinawa Chubu Hospital, Okinawa, Japan

## Abstract

**Background:**

We investigated the views of newly graduating physicians on their preparedness for postgraduate clinical training, and evaluated the relationship of preparedness with the educational environment and the pass rate on the National Medical Licensure Examination (NMLE).

**Methods:**

Data were obtained from 2429 PGY-1 physicians-in-training (response rate, 36%) using a mailed cross-sectional survey. The Dundee Ready Education Environment Measure (DREEM) inventory was used to assess the learning environment at 80 Japanese medical schools. Preparedness was assessed based on 6 clinical areas related to the Association of American Medical Colleges Graduation Questionnaire.

**Results:**

Only 17% of the physicians-in-training felt prepared in the area of general clinical skills, 29% in basic knowledge of diagnosis and management of common conditions, 48% in communication skills, 19% in skills associated with evidence-based medicine, 54% in professionalism, and 37% in basic skills required for a physical examination. There were substantial differences among the medical schools in the perceived preparedness of their graduates. Significant positive correlations were found between preparedness for all clinical areas and a better educational environment (all p < 0.01), but there were no significant associations between the pass rate on the NMLE and perceived preparedness for any clinical area, as well as pass rate and educational environment (all p > 0.05).

**Conclusion:**

Different educational environments among universities may be partly responsible for the differences in perceived preparedness of medical students for postgraduate clinical training. This study also highlights the poor correlation between self-assessed preparedness for practice and the NMLE.

## Background

One of the principal roles of medical schools is to equip newly graduating physicians for postgraduate clinical training [1-3]. Lack of preparedness for this training has been linked to greater stress in junior physicians and may also lead to poorer quality of patient care [4]. To improve clinical skills that can increase readiness for postgraduate clinical training, undergraduate medical education in Japan has recently begun implementing the Model Core Curriculum. This is proposed to restructure all areas of undergraduate medical education by requiring the implementation of the core curriculum for 70% of an entire curriculum and unique curriculum for remaining 30% in each medical school in order to facilitate learning by active participation in clinical clerkships [5]. The core curriculum emphasizes clinical practice through use of a problem-based learning (PBL), organ-based integrated curriculum (rather than a department-based approach), and implementation of the objective structured clinical examination (OSCE) [6].

In UK studies, self-perceived preparedness for postgraduate clinical training was found to differ substantially among medical schools [1,2]. Crucial factors influencing these differences may include a good educational environment for medical students, curricular changes with greater attention to preparedness for practice, adherence to modern educational theory, integration of basic and clinical sciences, and appropriate selection of students who will be successful medical graduates [7]. However, the extent, to which newly graduating physicians in Japan are prepared for postgraduate clinical training and differences among medical schools in terms of preparedness, has not been evaluated.

Preparedness for postgraduate clinical training of medical students is likely to depend on the engagement of medical students for learning clinical medicine. Educational environment is now considered as one of the most important factors determining engagement of medical students [8,9]. The educational environment is also considered to be the most significant manifestation and conceptualization of a curriculum, since it embraces everything that happens in a medical school [10]. Factors such as self-perceptions of learning, self-perceptions of teachers, academic self-perceptions, self-perceptions of atmosphere, and social self-perceptions are currently considered as major domains that comprise the educational environment of medical schools [11]. For instance, competitive, authoritarian, stressful, or intimidating environments may de-motivate students and weaken their engagement for learning clinical medicine. Environments that are collaborative, collegial, and supportive may enhance greater engagement of medical students for learning clinical medicine and the greater engagement may lead to improved preparedness for postgraduate clinical training. Thus, it is important to investigate whether a better environment is likely to help graduating physicians to be better prepared for postgraduate clinical training.

Preparedness for postgraduate clinical training could be assessed by different measures and self-perceived preparedness may be one for conducting nationwide studies like the previous UK report [1]. Thus, in the current study, we first sought the views of newly graduating physicians on their preparedness for postgraduate clinical training to obtain an understanding of this preparedness at different medical schools. Next, we evaluated the relationship between the educational environment and students' self-perceived preparedness at all 80 medical schools in Japan. We also evaluated the relationship between the pass rate on the National Medical Licensure Examination (NMLE) and self-perceived preparedness for postgraduate clinical training.

## Methods

### Subjects

In November 2008, a cross-sectional survey was mailed to 6725 1^st^-year resident physicians at 427 teaching hospitals with five or more 1^st^-year resident physicians, including 80 university hospitals and 347 non-university teaching hospitals throughout Japan. The program director at each hospital was asked to encourage the residents to complete the self-administered questionnaire. The academic calendar in Japan starts on April 1 and ends on March 31 of the following year; thus, the survey was conducted at the midpoint (November) of the 2008 academic year. We obtained ethical approval for the study from the ethics committee of St. Luke's International Hospital (the institution that the principal investigator was affiliated with at the time of the study) in Tokyo, Japan.

### Survey Contents

Data collected in the survey included the demographics of the respondents and their responses to the Dundee Ready Education Environment Measure (DREEM) inventory, which were used to assess the learning environment of the medical schools from which the respondents had graduated [11]. The earlier Japanese version of the DREEM was developed by Nishigori et al [12,13]. The original British version of the DREEM is in the public domain and was translated forward and backward by native-English speakers and native Japanese speakers, respectively, for development of a version for this study, the item translation of which was similar to that of the earlier version [12,13]. The DREEM inventory consists of 50 items, each of which is scored from 1 to 4 on a Likert scale, giving a maximum score of 200. A score of 100 or lower indicates a poor environment for learning medicine. The five DREEM domains include students' perception of learning, perception of teachers, academic self-perceptions, perception of atmosphere, and social self-perceptions. The DREEM has been validated as a measure of learning environment effectiveness in several previous studies [8,14].

The survey also included six questions related to preparedness for clinical competencies taken from the Association of American Medical Colleges Graduation Questionnaire (AAMC-GQ) after forward and backward translation into Japanese, for which we received permission from the AAMC. This questionnaire has been validated in several studies [15-17]. The questions represented a broad range of competencies for the following six clinical areas: 1) general clinical skills, 2) basic knowledge of diagnosis and management of common conditions, 3) communication skills, 4) skills for applying evidence-based medicine to clinical care, 5) professionalism, and 6) basic skills for physical examination. Participants rated their confidence in each of these areas using a five-point Likert scale: 1 (strongly agree), 2 (agree), 3 (neither agree nor disagree), 4 (disagree), and 5 (strongly disagree).

### Data Analysis

Participant responses were grouped by university that they graduated from. Spearman correlation coefficients and linear regression were used for analysis of relationships between the mean DREEM score for each medical school and the proportion of respondents from that school with confidence in their preparedness for each of the six clinical skills (as indicated by an answer of "strongly agree" or "agree"). Similar analyses were performed for the relationships between the pass rates on the 2008 NMLE (data were obtained from the Ministry of Health, Welfare and Labor) and the proportion of respondents with confidence in their preparedness. Individual performance scores were not available for analysis. Statistical analyses were conducted using SPSS 15.0J (Tokyo, Japan), with a two-tailed value of p < 0.05 considered to be statistically significant.

## Results

A questionnaire was sent to 6725 1st-year residents in 427 teaching hospitals in November 2008. A total of 2429 PGY-1 physicians-in-training (927 women, 38%) completed the questionnaire (response rate, 36%). Among the 80 medical schools from which the participants in the survey had graduated, the mean DREEM scores ranged from 95 to 137 (pooled mean 112; median 111; standard deviation 7.2). Three medical schools had a mean DREEM score of less than 100. The highest mean DREEM score was 137 (Table 1). The five subscales of the DREEM were highly inter-correlated (Table 2).

**Table 1:** DREEM score and confidence in preparedness for postgraduate training in six clinical areas in 80 medical schools (DREEM = Dundee Ready Educational Environment Measure, EBM = evidence-based medicine, PE = physical examination)

Rank by	Respondents	DREEM	Proportion of those confident in preparedness					
DREEM	n	Score (mean)	General	Basic knowledge	Communication	EBM	Professionalism	PE
1	30	137	0.433	0.433	0.733	0.333	0.800	0.700
2	48	134	0.250	0.396	0.667	0.271	0.833	0.604
3	33	125	0.273	0.394	0.636	0.242	0.667	0.576
4	27	125	0.074	0.259	0.444	0.185	0.481	0.333
5	18	124	0.167	0.556	0.500	0.389	0.667	0.500
6	12	124	0.167	0.500	0.417	0.417	0.750	0.667
7	27	124	0.111	0.407	0.407	0.333	0.667	0.407
8	21	123	0.381	0.286	0.571	0.190	0.571	0.429
9	45	121	0.156	0.156	0.556	0.378	0.556	0.533
10	31	121	0.387	0.387	0.677	0.097	0.645	0.484
11	28	120	0.321	0.321	0.571	0.393	0.679	0.571
12	9	120	0.333	0.222	0.444	0.333	0.444	0.667
13	30	119	0.333	0.367	0.667	0.333	0.633	0.433
14	27	119	0.111	0.333	0.444	0.333	0.630	0.444
15	29	119	0.138	0.241	0.586	0.103	0.655	0.414
16	15	118	0.000	0.267	0.400	0.133	0.733	0.400
17	23	118	0.174	0.217	0.478	0.174	0.652	0.391
18	30	117	0.400	0.500	0.767	0.233	0.700	0.600
19	34	116	0.147	0.324	0.471	0.206	0.471	0.324
20	38	116	0.026	0.289	0.263	0.132	0.421	0.211
21	42	116	0.286	0.310	0.643	0.214	0.524	0.452
22	19	115	0.368	0.263	0.526	0.105	0.421	0.421
23	36	115	0.333	0.333	0.472	0.278	0.444	0.444
24	23	114	0.087	0.130	0.435	0.087	0.609	0.348
25	20	114	0.200	0.250	0.500	0.100	0.750	0.250
26	21	114	0.238	0.333	0.714	0.190	0.667	0.667
27	30	114	0.100	0.267	0.567	0.200	0.533	0.300
28	24	114	0.250	0.500	0.625	0.375	0.625	0.375
29	45	114	0.178	0.356	0.644	0.178	0.622	0.356
30	19	113	0.158	0.158	0.474	0.105	0.421	0.421
31	34	113	0.088	0.176	0.382	0.088	0.500	0.206
32	34	113	0.206	0.294	0.382	0.176	0.559	0.382
33	38	113	0.105	0.263	0.526	0.237	0.605	0.342
34	12	113	0.167	0.500	0.500	0.333	0.500	0.333
35	45	113	0.156	0.267	0.533	0.333	0.644	0.378
36	29	112	0.103	0.310	0.448	0.138	0.552	0.552
37	23	112	0.087	0.304	0.565	0.217	0.565	0.261
38	30	112	0.133	0.200	0.533	0.167	0.633	0.467
39	35	112	0.143	0.200	0.543	0.114	0.543	0.314
40	33	111	0.212	0.242	0.394	0.091	0.455	0.333
41	56	111	0.161	0.304	0.554	0.214	0.429	0.339
42	53	111	0.170	0.321	0.453	0.208	0.623	0.396
43	42	111	0.381	0.452	0.667	0.262	0.762	0.476
44	37	111	0.135	0.243	0.324	0.135	0.459	0.216
45	26	111	0.077	0.154	0.192	0.115	0.269	0.192
46	25	110	0.240	0.360	0.600	0.240	0.520	0.480
47	36	110	0.139	0.278	0.500	0.167	0.611	0.306
48	10	110	0.100	0.500	0.700	0.100	0.700	0.400
49	22	110	0.045	0.182	0.455	0.091	0.545	0.091
50	18	110	0.167	0.167	0.500	0.167	0.444	0.389
51	25	110	0.200	0.400	0.480	0.280	0.480	0.400
52	43	110	0.163	0.116	0.488	0.070	0.395	0.349
53	18	110	0.222	0.333	0.444	0.167	0.500	0.556
54	51	109	0.059	0.137	0.314	0.078	0.373	0.196
55	24	109	0.125	0.333	0.292	0.208	0.500	0.417
56	34	109	0.088	0.235	0.382	0.206	0.529	0.324
57	22	109	0.091	0.318	0.455	0.182	0.500	0.227
58	36	109	0.139	0.278	0.389	0.194	0.556	0.250
59	39	109	0.026	0.308	0.359	0.179	0.513	0.385
60	28	109	0.143	0.464	0.429	0.214	0.429	0.357
61	26	109	0.192	0.231	0.346	0.154	0.423	0.346
62	32	108	0.094	0.344	0.375	0.156	0.531	0.313
63	19	108	0.105	0.211	0.316	0.211	0.632	0.211
64	45	108	0.089	0.133	0.244	0.089	0.356	0.222
65	17	108	0.235	0.412	0.353	0.176	0.471	0.412
66	44	108	0.114	0.295	0.318	0.159	0.364	0.250
67	35	107	0.143	0.171	0.400	0.114	0.486	0.229
68	33	107	0.091	0.030	0.606	0.030	0.485	0.364
69	32	107	0.188	0.219	0.438	0.188	0.438	0.250
70	43	106	0.093	0.233	0.488	0.140	0.512	0.256
71	30	106	0.100	0.267	0.467	0.167	0.500	0.367
72	35	106	0.229	0.286	0.343	0.171	0.429	0.429
73	28	105	0.071	0.179	0.321	0.107	0.357	0.250
74	37	104	0.054	0.243	0.378	0.135	0.432	0.378
75	25	104	0.160	0.200	0.480	0.160	0.560	0.360
76	39	102	0.103	0.179	0.256	0.051	0.308	0.231
77	23	102	0.043	0.087	0.435	0.217	0.478	0.261
78	28	99	0.143	0.214	0.571	0.179	0.643	0.250
79	13	95	0.077	0.231	0.462	0.077	0.538	0.154
80	22	95	0.091	0.318	0.455	0.227	0.455	0.273

**Table 2:** Spearman correlation coefficients among total score and subscales of the DREEM*

	Total score	Perception of learning	Perception of teachers	Perception of atmosphere	academic self-perceptions	social self-perceptions
Total score	1	0.931	0.845	0.884	0.933	0.731
Perception of learning		1	0.712	0.757	0.926	0.592
Perception of teachers			1	0.736	0.720	0.583
Perception of atmosphere				1	0.753	0.665
academic self-perceptions					1	0.601
social self-perceptions						1

*P-values of all correlation coefficients are <0.01.

Of the 2429 respondents to the survey, 406 (17%) agreed or strongly agreed that they were confident that they had acquired the general clinical skills required to begin postgraduate training. Overall, confidence in preparedness for postgraduate training in other clinical areas was indicated by 686 respondents (29%) for basic knowledge of diagnosis and management of common conditions, 1173 (48%) for communication skills, 465 (19%) for skills for applying evidence-based medicine to clinical care, 1317 (54%) for professionalism, and 904 (37%) for basic skills for physical examination.

Based on data for each school, the proportions of participants in the survey who were confident in their preparedness for postgraduate training ranged from 0% to 43% for general clinical skills; from 3% to 56% for basic knowledge of diagnosis and management of common conditions; from 19% to 77% for communication skills; from 3% to 42% for skills for applying evidence-based medicine to clinical care; from 27% to 83% for professionalism; and from 9% to 70% for basic skills for physical examination (Table 1).

**Figure 1:**
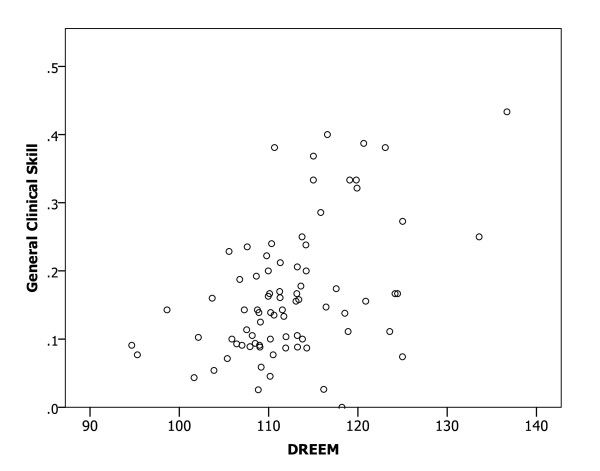
The DREEM score and confidence in preparedness for postgraduate training in general clinical skills in 80 medical schools (DREEM = Dundee Ready Educational Environment Measure).

**Figure 2:**
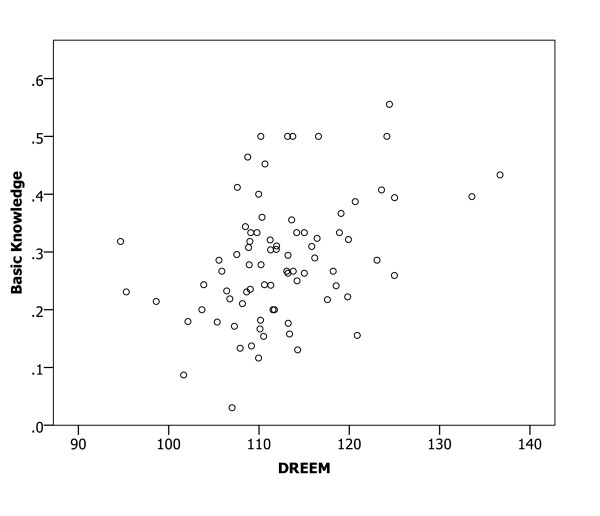
The DREEM score and confidence in preparedness for postgraduate training in basic knowledge of diagnosis and management of common conditions in 80 medical schools (DREEM = Dundee Ready Educational Environment Measure).

**Figure 3:**
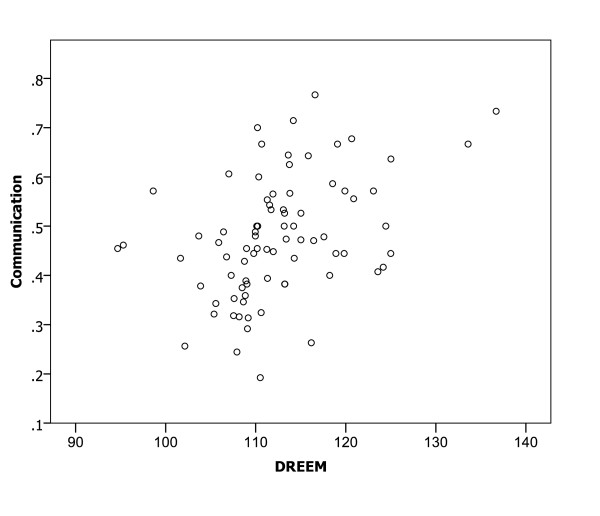
The DREEM score and confidence in preparedness for postgraduate training in communication skills in 80 medical schools (DREEM = Dundee Ready Educational Environment Measure).

**Figure 4:**
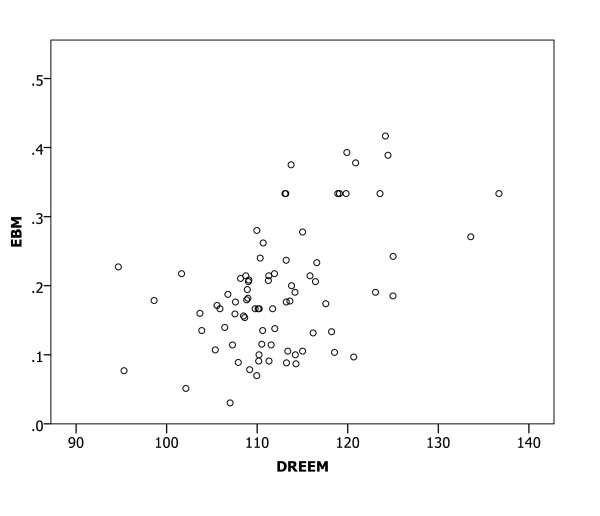
The DREEM score and confidence in preparedness for postgraduate training in skills for applying evidence-based medicine to clinical care in 80 medical schools (DREEM = Dundee Ready Educational Environment Measure, EBM = evidence-based medicine).

**Figure 5:**
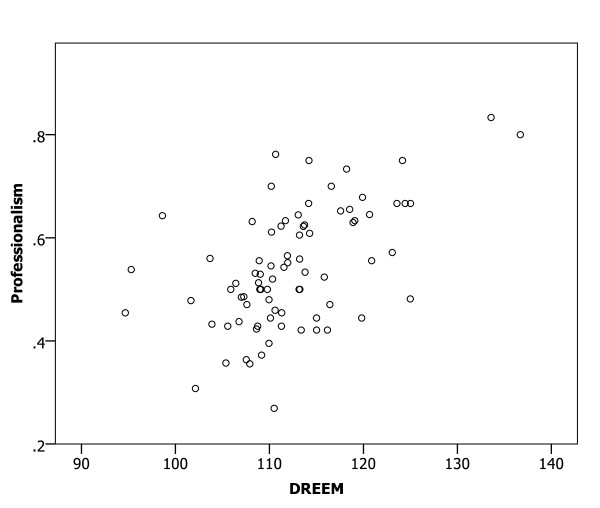
The DREEM score and confidence in preparedness for postgraduate training in professionalism in 80 medical schools (DREEM = Dundee Ready Educational Environment Measure).

**Figure 6:**
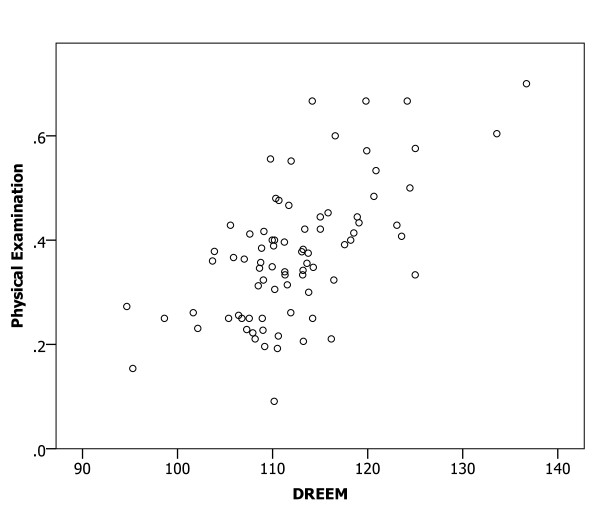
The DREEM score and confidence in preparedness for postgraduate training in basic skills for physical examination in 80 medical schools (DREEM = Dundee Ready Educational Environment Measure).

A Spearman correlation analysis of the relationships between mean DREEM scores for medical schools and the proportions of participants with confidence in preparedness indicated significant relationships (all p < 0.01) in all six clinical areas (Figures 1, 2, 3, 4, 5 and 6), with positive coefficients of 0.425 for general clinical skills (R^2 ^= 0.218), 0.377 for basic knowledge of diagnosis and management of common conditions (R^2 ^= 0.160), 0.452 for communication skills (R^2 ^= 0.169), 0.402 for skills for applying evidence-based medicine to clinical care (R^2 ^= 0.236), 0.522 for professionalism, R^2 ^= 0.294), and 0.568 for basic skills for physical examination (R^2 ^= 0.393). A Spearman correlation analysis of the relationships between each of all five DREEM subscales for medical schools and the proportions of participants with confidence in preparedness also indicated significant relationships (all p < 0.05) in all six clinical areas.

The pass rates for the 2008 NMLE ranged from 85% to 99%. A Spearman correlation analysis of the relationships between these pass rates and the proportion of participants with confidence in their preparedness indicated no significant relationship in any of the six clinical areas, with coefficients of 0.035 for general clinical skills (p = 0.758), 0.039 for basic knowledge of diagnosis and management of common conditions (p = 0.733), 0.123 for communication skills (p = 0.277), -0.077 for skills for applying evidence-based medicine to clinical care (p = 0.496), 0.078 for professionalism (p = 0.492), and -0.012 for basic skills for physical examination (p = 0.917). The mean DREEM scores and pass rates for the universities also showed no significant relationship (Spearman correlation coefficient = 0.209, p = 0.063).

## Discussion

The current study showed that only 17% of our responding newly graduated physicians from all medical schools in Japan felt prepared by their schools in general clinical skills for their first postgraduate clinical training. Perceived preparedness for other clinical areas was similarly unsatisfactory. These results are in line with those of our recent pilot report indicating that Japanese medical graduates perceive that they are not well prepared clinically to start working as physicians-in-training, and that self-reported preparedness is significantly lower in Japanese graduates than in their US counterparts [18]. However, because of a limited ability to accurately self-assess among professionals [19], external assessment is needed for evaluating a real difference in preparedness between Japan and US.

The Model Core Curriculum has been introduced in undergraduate medical education throughout Japan. But medical schools in Japan vary greatly in the extent to which they have adopted the Model Core Curriculum, and there are still considerable differences in the duration and contents of clinical clerkships among medical schools [20,21]. In many schools, students have limited opportunities to examine and interact with patients and little direct responsibility for patient care [22]. In this study, we found substantial differences among Japanese medical schools in self-perceived preparedness of their graduates, with significant positive correlations between self-assessed preparedness for all clinical areas and a better educational environment. These findings suggest that differences in educational environments among medical schools may be partly responsible for the differences in perceived preparedness of graduates for postgraduate clinical training. The implications of this finding and the need for improvement of the educational environment for medical students should be of concern to medical educators.

Engagement is a crucial step in learning that depends not only on the motivation and learning style of students, but also on the environment or "climate" in which the learning is taking place [23,24]. Genn and Harden suggested that the educational environment is the soul and spirit of the medical curriculum and that establishing an effective environment is the most important single task of medical educators [10,25]. Components of the educational environment related to the curriculum include the style (e.g., PBL vs. a traditional approach) and quality of teaching, signposting and clarity of the process, outcomes, assessment, and support mechanisms for students. Components of the educational environment related to individual teachers include teaching style, enthusiasm, physical environment, and role modeling [23]. Thus, our finding of significant positive correlations between perceived preparedness for all clinical areas and a better educational environment in medical schools reflects the importance of this environment in affecting the extent of engagement of medical students. Further research is needed to determine the components in an educational environment that are most strongly related to preparedness.

We found no significant association between the pass rate on the NMLE and perceived preparedness for any of the six clinical areas addressed in our survey. Traditionally in Japan, medical students have been required to attend lectures that were aimed at equipping the students to pass the NMLE (a paper-only test with a major emphasis on cognitive domains), even in the last two years of clinical training in a six-year medical school program. Some schools still emphasize this traditional curriculum, with little emphasis on the new Model Core Curriculum [20,21]. However, the result of our study showing lack of association between the pass rate on the NMLE and perceived preparedness may indicate poor predictive validity of students' perceived preparedness for their first postgraduate clinical training against the NLME outcomes. This may also reflect relative insensitivity for identifying any association between the NMLE and perceived preparedness when using data about the pass rate. Thus, this result should be cautiously interpreted and there would be a need for further investigations such as a study using actual scores of the exam rather than pas-fail rates.

If our results would be confirmed using actual scores of the NMLE, a change of the contents and assessment method of the NMLE, such as introduction of clinical skills assessment or an OSCE-type test, could be considered for improving preparedness by helping schools to focus their training on this aspect. Another possibility for a change of the exam would be to administer the cognitive domain tests of the exam prior to entry into the clinical clerkship period in the last two years of medical school, similar to the United States Medical Licensing Exam (USMLE) Step 1.

There are several limitations in this study. First, our results might have been influenced by sampling bias, since the response rate was relatively low and many residents may have been too busy to respond to the survey. As we have conducted a cross-sectional survey for 1st-year resident physicians at teaching hospitals with five or more 1st-year resident physicians, we did not obtained data from teaching hospitals with four or less 1st-year resident physicians. Thus, we did not know the range of response rates across schools and gender ratio in all PGY-1 population. However, our sample size was large and we were able to examine a nationwide sample. Although Japanese Ministry of Health, Welfare and Labor does not open data related to individual residents, based on data about 46,800 medical students in 2006, 33% were women [26]. So our sample (38% were women) seemed not much different from the total population.

Second, our survey only investigated each resident's perception of their preparedness, rather than using clinical or objective assessment of preparedness. Thus, we cannot determine the differences in outcomes between universities objectively. Finally, because of the cross-sectional observational nature of the survey, we are unable to determine a causal link between the educational environment and preparedness, since confounding factors such as personal characteristics of students that may be related to both environment and preparedness may have influenced the results.

## Conclusions

In summary, Japanese medical graduates perceive that they are not well prepared clinically to start working as physicians-in-training. There were substantial differences among Japanese universities in the perceived preparedness of their graduates. Significant positive correlations were found between perceived preparedness for all clinical areas and a better educational environment, but there was no significant association between the pass rate on the National Medical Licensure Examination and perceived preparedness for any clinical areas. The link between self-perceived and independently observed preparedness needs to be established. Then, further research will be required to evaluate the most important components in the educational environment that influence preparedness. Japanese medical schools may need to consider establishment of minimum requirements for clinical competencies for their medical graduates.

## Competing interests

The authors declare that they have no competing interests.

## Authors' contributions

YT conceived of the study, analyzed the data, and drafted the manuscript. EG, JO, JJ, and FO participated in the design of the study and assisted writing of the manuscript. HO participated in its design and coordination. MS, KS, YI, SO, OT, and TF participated in the design of the study and assisted the data analysis. All authors read and approved the final manuscript.

## Pre-publication history

The pre-publication history for this paper can be accessed here:

http://www.biomedcentral.com/1472-6920/10/35/prepub

## References

[B1] Goldacre MJ Lambert T Evans J Turner G Preregistration house officers' views on whether their experience at medical school prepared them well for their jobs: national questionnaire survey BMJ 2003 326 7397 1011 1012 10.1136/bmj.326.7397.1011 12742922 PMC154758

[B2] Illing J Morrow G Kergon C Burford B Spencer J Peile E Davies C Baldauf B Allen M Johnson N How prepared are medical graduates to begin practice? A comparison of three diverse UK medical schools. Final summary and conclusions for the GMC Education Committee GMC Annual Report; London 2008

[B3] GMC Tomorrow's Doctors 2009 http://www.gmc-uk.org/education/undergraduate/tomorrows_doctors_2009.asp

[B4] Paice E Rutter H Wetherell M Winder B McManus IC Stressful incidents, stress and coping strategies in the pre-registration house officer year Med Educ 2002 36 1 56 65 10.1046/j.1365-2923.2002.01101.x 11849525

[B5] Abe Y The Model Core Curriculum Igaku-Kyoiku (Japanese) 2002 33 77 82

[B6] Teo A The current state of medical education in Japan: a system under reform Medical Education 2007 41 3 302 308 10.1111/j.1365-2929.2007.02691.x 17316216

[B7] Cave J Goldacre M Lambert T Woolf K Jones A Dacre J Newly qualified doctors' views about whether their medical school had trained them well: questionnaire surveys BMC Med Educ 2007 7 38 10.1186/1472-6920-7-38 17945007 PMC2203980

[B8] Whittle SR Whelan B Murdoch-Eaton DG DREEM and beyond; studies of the educational environment as a means for its enhancement Educ Health (Abingdon) 2007 20 1 7 17647175

[B9] Bassaw B Roff S McAleer S Roopnarinesingh S De Lisle J Teelucksingh S Gopaul S Students' perspectives on the educational environment, Faculty of Medical Sciences, Trinidad Med Teach 2003 25 5 522 526 10.1080/0142159031000137409 14522676

[B10] Genn JM AMEE Medical Education Guide No. 23 (Part 1): Curriculum, environment, climate, quality and change in medical education-a unifying perspective Med Teach 2001 23 4 337 344 10.1080/01421590120063330 12098379

[B11] Roff S The Dundee Ready Educational Environment Measure (DREEM)--a generic instrument for measuring students' perceptions of undergraduate health professions curricula Med Teach 2005 27 4 322 325 10.1080/01421590500151054 16024414

[B12] Nishigori H Yoshida I DREEM, PHEEM, ATEEM and STEEM in Japanese Igaku-Kyoiku (Japanese) 2006 37 2 4 14

[B13] Nishigori H Nishigori M Yoshimura H DREEM, PHEEM, ATEEM and STEEM in Japanese Med Teach 2009 31 6 560 10.1080/01421590903136488 19811172

[B14] de Oliveira Filho GR Vieira JE Schonhorst L Psychometric properties of the Dundee Ready Educational Environment Measure (DREEM) applied to medical residents Med Teach 2005 27 4 343 347 10.1080/01421590500046387 16024418

[B15] Dial TH Lindley DW Predictive validity of specialty choice data from AAMC graduation questionnaire. Association of Medical Colleges J Med Educ 1987 62 12 955 958 3681935 10.1097/00001888-198712000-00001

[B16] Coates WC Crooks K Slavin SJ Guiton G Wilkerson L Medical school curricular reform: fourth-year colleges improve access to career mentoring and overall satisfaction Acad Med 2008 83 8 754 760 10.1097/ACM.0b013e31817eb7dc 18667890

[B17] Patel MS Lypson ML Davis MM Medical student perceptions of education in health care systems Acad Med 2009 84 9 1301 1306 10.1097/ACM.0b013e3181b17e3e 19707077

[B18] Obara H Tokuda Y Orlander JD Perceived Preparedness of Japanese Medical Graduates for Postgraduate Training: a Comparison to US Graduates 2008 Society of General Internal Medicine; Pittsburgh, USA

[B19] Balcetis E Dunning D Miller R Do collectivists "know themselves" better than individualists? Cross-cultural studies of the "holier than thou" phenomenon Journal of Personality and Social Psychology 2008 95 6 1252 1267 10.1037/a0013195 19025282

[B20] Sasaki H Arimura K Itoyama Y Kwak S Kira J Nakashima K Amano T Inoue K Uozumi T Kohara N Nationwide questionnaire study in "the Model Core Curriculum" and current status for the undergraduate education in neurology Rinsho Shinkeigaku(Japanese) 2008 48 8 556 562 10.5692/clinicalneurol.48.556 18939474

[B21] Onishi H Yoshida I Rapid change in Japanese medical education Med Teach 2004 26 5 403 408 10.1080/01421590412331270492 15369878

[B22] Tierney L Jr An experience in Japanese academic medicine Western Journal of Medicine 1994 160 2 139 8160464 PMC1022319

[B23] Hutchinson L Educational environment BMJ 2003 326 7393 810 812 10.1136/bmj.326.7393.810 12689981 PMC1125718

[B24] McAleer S Soemantri D Roff S Chaper 9. Educational environment A practical guide for medical teachers 2009 3 Edinburgh; New York: Elsevier Churchill Livingstone 64 72

[B25] Genn JM Harden RM What is medical education here really like? Suggestions for action research studies of climates of medical education environments Med Teach 1986 8 2 111 124 10.3109/01421598609010737 3762357

[B26] Kozu T Medical education in Japan Acad Med 2006 81 12 1069 1075 10.1097/01.ACM.0000246682.45610.dd 17122471

